# The manipulation of cell suspensions from zebrafish intestinal mucosa contributes to understanding enteritis

**DOI:** 10.3389/fimmu.2023.1193977

**Published:** 2023-05-12

**Authors:** Xuyang Zhao, Yuhang Liu, Jiayuan Xie, Lei Zhang, Qingsong Zhu, Lian Su, Cheng Guo, Heng Li, Guangxin Wang, Wanting Zhang, Yingyin Cheng, Nan Wu, Xiao-Qin Xia

**Affiliations:** ^1^ Institute of Hydrobiology, Chinese Academy of Sciences, Wuhan, China; ^2^ College of Fisheries and Life Science, Dalian Ocean University, Dalian, China; ^3^ College of Advanced Agricultural Sciences, University of Chinese Academy of Sciences, Beijing, China

**Keywords:** zebrafish, intestine, cell suspension, flow cytometry, SBMIE, transcriptome

## Abstract

**Background:**

Although zebrafish are commonly used to study intestinal mucosal immunity, no dedicated procedure for isolating immune cells from zebrafish intestines is currently available. A speedy and simple operating approach for preparing cell suspension from mucosa has been devised to better understanding of intestinal cellular immunity in zebrafish.

**Methods and results:**

The mucosal villi were separated away from the muscle layer by repeated blows. The complete deprivation of mucosa was done and evidenced by HE and *qPCR* results. Higher expression of both innate (*mpeg1*, *mpx*, and *lck*) and adaptive immune genes (*zap70*, *blnk*, *foxp3a*, and *foxp3b*) was revealed compared to cells obtained by typical mesh rubbing. The cytometric results also revealed that the tested operation group had a higher concentration and viability. Further, fluorescent-labelled immune cells from 3mo *Tg(lyz:DsRED2)*, *Tg(mpeg1:EGFP)*, *Tg(Rag2:DsRED)*, and *Tg(lck:EGFP)*, were isolated and evaluated for the proportion, and immune cells’ type could be inferred from the expression of marker genes. The transcriptomic data demonstrated that the intestinal immune cell suspension made using the new technique was enriched in immune-related genes and pathways, including *il17a/f, il22, cd59*, and *zap70*, as well as pattern recognition receptor signaling and cytokine-cytokine receptor interaction. In addition, the low expression of DEG for the adherent and close junctions indicated less muscular contamination. Also, lower expression of gel-forming mucus-associated genes in the mucosal cell suspension was consistent with the current less viscous cell suspension. To apply and validate the developed manipulation, enteritis was induced by soybean meal diet, and immune cell suspensions were analyzed by flow cytometry and qPCR. The finding that in enteritis samples, there was inflammatory increase of neutrophils and macrophages, was in line with upregulated cytokines (*il8* and *il10*) and cell markers (*mpeg1* and *mpx*).

**Conclusion:**

As a result, the current work created a realistic technique for studying intestinal immune cells in zebrafish. The immune cells acquired may aid in further research and knowledge of intestinal illness at the cellular level.

## Introduction

Fish mucosal immunity has developed as a prominent study topic in aquaculture over the last decade. Gut-associated lymphoid tissue (GALT) is one of the mucosa-associated lymphoid tissues (MALT) in fish that plays a vital function in treating foodborne antigens and balancing intestinal microbiota (1). Unlike mammalian gut-associated structured lymphoid tissues such as mesenteric lymph nodes and Peyer’s patches, fish GALT only contains the more widely dispersed immune cells in the intestinal epithelium.

As to the MALT studied in many fish species, such as goldfish (*Carassiusauratus*) (2), dogfish (*Scyliorhinuscanicula* L.) (3), bloch (*Channa Punctatus*) (4), and carp (*Cyprinuscarpio*L.) (5), there are no organized follicles. The crypt–villus tissue architecture in combination with rapid cell turnover enables the intestine to play an immune barrier role. Continuous tissue replacement in mammals is fueled by continually dividing stem cells at the bottom of crypts (6). Consequently, as in medaka (7) the germinal centers of fish GALT may also lie in the crypt of intestinal villi. Tissue-resident immune cells differ significantly from blood cells (8). In fish, gut immune cells are heterogeneous and changeable. The immune system of the fish gut also plays an important role in maintaining the immune barrier by developing tolerance to antigens from the diet and commensal bacteria, while providing an effective response to pathogens. Therefore, it is important to study how an immune response of the gut is triggered and what the outcomes are at the cellular level.

By light and electron microscopic observation, it has been found in goldfish that the epithelium contains a number of migrating leukocytes, including lymphocytes and lymphoblasts, macrophages and heterophils (2). According to the ontology study of carp intestinal immune cells, WCL38^+^ intraepithelial lymphocytes and WCL15^+^ macrophages were detected in both LP layers (9). Increasing experimental evidence suggests that fish lymphocytes may share developmental, morphological and functional features with mammalian innate lymphocytes. The intestine is the most important lymphoid tissue for T cells in fish. Purified intestinal T cells from sea bass (*Dicentrarchus labrax*) showed increased expression of RAG -1, TcRα, TcRγ, CD8α and CD4 (10).

The intestinal mucosa in fish consists of three compartments: Epithelium, lamina propria and muscularis mucosae. The epithelial cells in the IEL (intestinal epithelial layer) act as an immune barrier, and the goblet cells that secrete mucin could support the interaction between the mucosal surface and microorganisms. The eosinophil granule cells of fish, which resemble mammalian Paneth cells in their possession of lysozyme-containing granules (11), could also secrete granules. Below the IEL is the LP (lamina propria), which contains mainly lymphocyte- and macrophage-like cells (3) that can defend against invaders (antigens). In contrast, there are few immune cells in the muscularis mucosae, only with resident macrophages (12).

The dynamic recruitment or infiltration of immune cells in the gut is of great importance in both physiology and pathology. Signals originating from both innate and adaptive cells regulate the activation of innate lymphocytes in non-lymphoid tissues, contributing to mucosal inflammation and disease (13). In zebrafish (*Danio rerio*), lymphocytes colonize the gut as early as 5 days after fertilization and increase in number during the inflammatory process (14). The extent of accumulation and the number of immune cells infiltrating the LP and the IEL increased in fish when exposed to pathogens (such as *Aeromonas hydrophila*) or food allergens.

In aquaculture, the substitution of fishmeal with vegetable proteins such as soymeal brings with it many antinutrients, leading to disruption of the intestinal villi both structurally and functionally (15). Because the zebrafish is genetically defensible and optically accessible, it has been used to study the cellular and molecular mechanisms of fish diseases (16). The zebrafish has been used to study the pathology of enteritis (17), intestinal dysplasia (18), IBD triggered by chemicals (such as TNBS and DSS) (19, 20) and food-borne enteritis (21–23). Since food-borne enteritis not only causes mucosal injury but also accelerates metaflammation in aquaculture (24), soybean meal-induced enteritis (SBMIE) was widely to modelling enteritis for both inflammatory infiltration of immune cells and typical Th17 response in the gut (14) and fatty deposits in the liver (23).

However, the isolation of GALT’s immune cells has been performed in several cultured fish species, such as rainbow trout (*Oncorhynchus mykiss*) (25), carp and turbot (*Scophthalmus maximus*) (26). In order to analyze the proportion of immune cells in the gut, the preparation of single cells is a crucial part of flow cytometric analysis of the gut (27). However, the preparation of cell suspensions from the zebrafish gut is not yet feasible due to the more minute and fragile structure compared to cultured fish species. Enzymatic digestion would compromise cell viability in the thin mucosa of the zebrafish gut, and mechanical dissociation with tissue dissociation instruments is difficult to control in very small tissue.

According to our previous findings in the SBMIE zebrafish model, the cells of the innate immune system (neutrophils and macrophages) responded immediately and actively in the intestine to food-induced enteritis. To facilitate the study of the regulatory mechanism of the intestinal immune system at the cellular level, the isolation of intestinal immune cells from zebrafish, particularly in fluorescently labelled lines, was performed by optimizing the manipulation. Subsequently, transcriptomic analysis was performed after cell sorting by flow cytometry to verify cell populations. In addition, the expression of inflammatory genes was verified in the SBMIE model. The current working and analysis method for zebrafish is useful to understand the gut immune response at the cellular and molecular level, both in healthy and diseased animals.

## Materials and methods

### Zebrafish and husbandry

The wild-type AB zebrafish and transgenic lines, including *Tg(lyz:DsRED2)* (labeling neutrophils (28), https://zfin.org/ZDB-TGCONSTRCT-071109-3), *Tg(rag2:DsRed)* (labeling lymphocytes (29), http://zfin.org/ZDB-TGCONSTRCT-131022-4), *Tg(lck:EGFP)* (labeling T lymphocytes (30), https://zfin.org/ZDB-TGCONSTRCT-070117-48), *Tg(mpeg1:EGFP)* (labeling macrophages (31), http://zfifin.org/ZDB-TGCONSTRCT-120117-1),were purchased from the China Zebrafish Resource Center (CZRC). Then, the double labeled fish*Tg(lyz:DsRED2)*;*Tg(mpeg1:EGFP)* were obtained by crossing related strains. Zebrafish was maintained, raised, and reproduced as previously described (21).

### Diets and the feeding trail

The feeding of zebrafish larvae began with the feeding of *Paramecium caudatum* at 6 dpf (day post fertilization) until they were able to survive by feeding on live brine shrimp. Thereafter, the zebrafish were fed brine shrimp for at least 3 months. To model SBMIE (soybean meal-induced enteritis) in the zebrafish, the experimental diets (soybean meal diet (50SBM) or fish meal diet) were fed as previously described (21, 32). 3-month-old zebrafish (n = 150) were kept in 6 tanks (3L volume). The fish were fed fishmeal for a fortnight. Afterwards, the fish were fed twice daily with soybean meal or fish meal (as control) for one week.

### Sampling

For sampling, all reagents and tools were pre-sterilized or disinfected with 75% ethanol. Zebrafish were euthanized with 0.2mg/ml tricaine methanesulfonate (MS -222, Sigma-Aldrich) solution after fasting for 24 hours to promote intestinal emptying (33). The tail of the zebrafish was cut off with a sharp scalpel and then the body dissected to obtain the internal organs. Separate the whole intestine with its orientation, stretch it out and roll it gently on the absorbent paper to loosen the sticky mesentery (34) with tweezers. The operation to divide the zebrafish intestine was performed as previously described (35, 36), with some modifications. The hindgut tissue (last third segment) was prepared for haematoxylin-eosin staining (HE), meanwhile cell samples were collected for quantitative real-time PCR (qPCR) or preparation of gut cell suspensions for flow cytometry analysis (e.g. fluorescence activated cell sorting, FACS). Blood was collected from the tail and 0.1 ml of 10mg/ml heparin sodium was added to prevent clotting. Single cell suspensions from the internal tissues, including intestine, liver, kidney and spleen, were obtained by filtering the minced tissue through a 300 mm nylon mesh (37). For liver, kidney and spleen, the tissue was rubbed several times in a small amount of cold PBS with 1% FBS (fetal bovine serum) on the nylon mesh before filtration.

### Histological analysis

The hindgut was used for HE staining. One half was fixed directly with paraformaldehyde, while the other half was used to prepare a cell suspension. The remaining tissue after preparing the intestinal cell suspension was also fixed with paraformaldehyde. All samples were cut into sections (10μm) and stained as previously described (23).

### Manipulation of single cell suspension from zebrafish intestinal mucosa

First, the hindgut (last third of the intestine) were dissected from adult zebrafish (about 3 mouth-old), and then was lengthwise cutting with a micro-eye scissor (Zrbiorise). The intestinal tissue was transferred to 400μl of cold PBS (4°C) in a 1.5ml pre-cold microtube (on ice) with vortexing (on the Vortex Mixer, Shanghai Huxi, WH-2) for 2 seconds to dissociate the attaching mucus. The tissue was then transferred to 1.5ml cold PBS (4°C) containing 1% FBS (fetal bovine serum). Meanwhile, 200µl micropipette was adjusted to the volume of 150µl, and the tip was cut off at the front end with a sharp scalpel. This was to ensure that the intestinal tissue could easily pass though. Finally, to release the immune cells in epithelial and LP layers, the hindgut tissue was blowing repeatedly, until the tissue looked semitransparent. Additionally, for the liver, kidney and spleen of the zebrafish, this step ended with the tissue being minced without any obvious tissue fragments being present.

Only the cell suspension was kept to be filtered through a 300 mesh nylon sieve and lightly centrifuged (600 g for 5 min at 4°C). After discarding the supernatant, the cells were resuspended in the flask in an additional 300μl of cold PBS containing 1% FBS. The cells could be washed a second time by gentle centrifugation. 300μl cold PBS with 1% FBS was added to dilute the suspension and prevent the cells from becoming entangled by mucus secretion. During all steps, the cells were kept on ice at all times to ensure cell viability. Finally, the cells could be immediately analyzed with a cytometer.

### Evaluating cell viability, morphology and fluorescence

The properties of the intestinal cell suspension obtained, including cell viability, morphology and fluorescence, were evaluated using both an automatic cell counter (Countstar) and a fluorescence microscope (Olympus). To check cell viability, acridine orange/propidium iodide (AO/PI) was used to label dead cells with red fluorescence, while living cells were genetically visualized with green fluorescence. 10 ul AO/PI were added to the same volume of homogenised cell suspension. After mixing, the cell suspension was immediately transferred to the slide to carefully observe the cell size and quantity. To determine the fluorescence properties, the cell suspension samples of the genetically fluorescently labelled transgenic strains were examined under the fluorescence channels.

### RNA extraction and qPCR analysis

Total RNA extraction of intestinal tissue and mucosal cells after centrifugation was performed with Trizol (Invitrogen). RNA was quantified using Nanodrop2000 (Thermo Fisher) and its integrity was checked on a 1% agarose gel. cDNA was synthesized from RNA samples (600 ng) using HiScript^®^ II QRT SuperMix for qPCR with gDNA wiper (Vazyme). Quantitative real-time PCR (qPCR) was performed on a Bio-Rad CFX Maestro™ (Bio-Rad) instrument with 6ng cDNA per well using Hieff^®^ qPCR SYBR^®^ Green Master Mix (Yeasen). The run was 95°C for 5 minutes, then 95°C for 10 seconds, 60°C for 15 seconds, 72°C for 60 seconds with 41 cycles. Each reaction was performed in triplicate. The qPCR analysis was performed according to previously published procedures (38, 39) with *rpl13a* serving as the endogenous control. The average ΔCt value was calculated by subtracting the control ΔCt value from the treated ΔCt value. The relative amount of mRNA was calculated as 2^-ΔΔCt^. The primers used in the current study were partially cited from published articles or designed and tested using the primer BLAST in NCBI. The primers used are summarized in **Table 1**.

**Table 1:** All primers used for qPCR analysis of enteritis related genes.

Gene	Genbank ID	Forward primer	Reverse primer
*rpl13a*	NC_007128.7	TCTGGAGGACTGTAAGAGGTATGC	AGACGCACAATCTTGAGAGCAG
*lck*	NC_007128.7	GCCGAAGAAGATCTCGATGGT	TCCCCATGTTTACGTATTTTGTCG
*mpx*	NC_007121.7	CTACATGGCACAAACGCTGAG	CTCGTCTTGAGTGAGCAGGTT
*mpeg1*	NC_007119.7	TGCGGCACAATCGCAGTCCA	ACAGCAAAACACCCATCTGGCGA
*foxp3a*	NC_007119.7	GCCTCCATGATACGATGGGCAAT	CCTTCCTTCAACACGCACAA
*foxp3b*	NC_007119.7	AGACAACGGCTGTCAACTAA	TGAAGAAACTGCATTCGCTG
*cd4-1*	NC_007127.7	CTGACAATCAACAGGAACCC	TCTTGCTAATACATGTTGCTCA
*blnk*	NC_007124.7	GGACAGGTTCACACTCATTAC	GTCTCTTGCTGGAACTTTGG
*zap.70*	NC_007119.7	AGATCTGGCTGCTCGTAATG	AAAATGAATGCACTCTGGCG
*il-10*	NC_007122.7	CACTGAACGAAAGTTTGCCTTAAC	TGGAAATGCATCTGGCTTTG
*il-1β*	NC_007121.7	TGGACTTCGCAGCACAAAATG	GTTCACTTCACGCTCTTGGATG
*nf-kb*	NC_007125.7	GCGCTTTTCTGAATCCTACG	TGCCCAGTCTGTCTCCTTCT
*il-8*	NC_007112.7	TGTGTTATTGTTTTCCTGGCATTTC	GCGACAGCGTGGATCTACAG

### Flow cytometry analysis

To count dead cells, after centrifugation and removal of supernatant, cells were diluted with 100 ul of cold 1×DAPI (Coolaber) for 5 minutes. The suspension was then immediately diluted with 300 μl cold PBS containing 1% FBS to prevent cell adhesion caused by mucus formation and then filtered through a 300 mesh nylon to prevent clogging of the tubes for the flow cytometer. The specific workflow refers to the previously published protocol (40). Briefly, the definition of the different cell types was done by a large number of events, and the identification of live or dead cells was visualized by iodopropylidine (PI) staining. On the flow cytometry (CytoFLEX S), the parameters, such as the number of cells, the region of interest, voltage and balance, selection of detectors, were set to analyze all samples. In addition, the cell suspension stained and filtered with DAPI was kept on ice during all manipulations to ensure cell viability.

### Sorting of fluorescent labeled immune cells from multiple organs

After staining with DAPI and filtration, the samples of cell suspension from multiple organs, including blood, gut, liver, kidney and spleen, were analyzed using flow cytometry. The intestinal mucosal cell suspension from four zebrafish were mixed and sorted for immune fluorescence-labeled cells on a high-speed flow sorting cytometry (Becton Dickinson). Two operation repetitions were carried out for each sample.

### Library preparation and sequencing for transcriptomic data

Transcriptomic analysis was used to systemically disclose the expression of intestinal RNA (n = 3) in both the intestinal tissue (IT group) and current prepared intestinal mucosa cell suspension (IMC group). The process for preparing the gene library and sequencing the transcriptome was done according to previously described methods (41). In brief, sequencing libraries were prepared using the NEBNextRUltra™ RNA Library Prep Kit for Illumina R (NEB, USA), and the quality of the libraries was determined using the Agilent Bioanalyzer 2100 system. On an Illumina platform (NovaSeq 6000), the library preparations were sequenced and 150bp paired-end reads were produced.

### Transcriptome assembly, DEG analysis and functional annotation

We used Hisat2 (42) to map the clean reads to the zebrafish genome (GRCz11, https://www.ncbi.nlm.nih.gov/genome/?term=txid7955[orgn]). The lots of reads that were mapped was counted by verse (version 0.1.5). Imputing the reads counts to DESeq2 (version 1.24.0) and analyze it DEGs. To select and analyze DEGs, we use DESeq2 (43) with | log2FoldChange | > 1 and padj 0.05. Then, for annotation, cluster Profiler (version 3.12.0) (44) was used to perform enrichment analysis of GO (Gene Ontology) terms and KEGG (Kyoto Encyclopedia of Genes and Genomes) pathways, with *p* < 0.05 considered significant enrichment. When the parameters used were not listed, default parameters were used. Afterwards, according to FishSCT (http://bioinfo.ihb.ac.cn/fishsct/), the immune cell, neuron, and muscle related marker genes were selected, to infer cell types. Further, the RPKM of related DEGs were applied in Visual Omics (http://bioinfo.ihb.ac.cn/visomics) (45), to generate a heatmap.

### Statistical analysis

For all data, statistical analyses and imaging were performed using the computer program GraphPad Prism version 8.0 (GraphPad Software Inc. CA, United States). For comparison between two group, data were analyzed with paired student’s T-test with qPCR data analysis and unpaired student’s T-test with others experiment’s results. The bar diagram represented mean ± SD. Differences were considered statistically significant at *p* < 0.05 (*), *p* < 0.01 (**), *p* < 0.001 (***), and *p* < 0.0001 (****).

## Results

### Improved operation for preparing intestinal cell suspension in zebrafish

**Figure 1:**
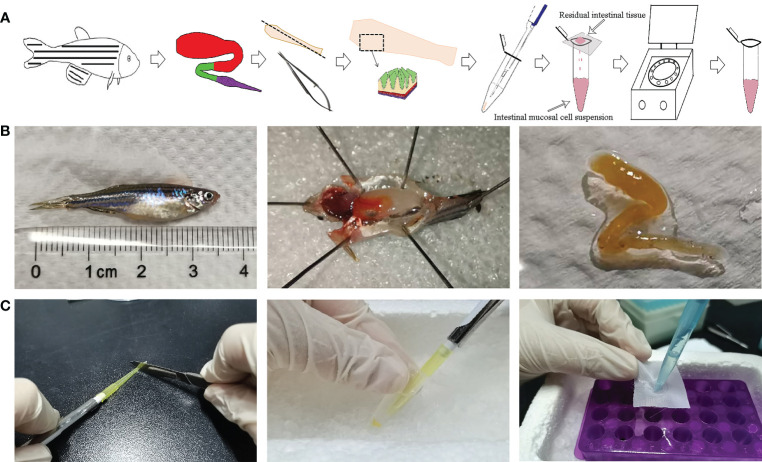
Operating processes for preparing cell suspension from zebrafish intestinal mucosa. **(A)** Schematic diagram of operating processes. **(B)** Photographs of the detail operation, including dissection, intestinal separation, blowing and filtering.

After anaesthesia, the intestine of the zebrafish was removed to prepare an intestinal cell suspension, as the intestine is relatively simple in structure and there is no submucosa between the mucosa and muscle. The primary steps are shown in (**Figure 1A**), so the immune cells in the zebrafish intestinal mucosa were released by the currently developed blowing method (**Figure 1B**). Specifically, first, an adult zebrafish (with a length of approximately 3.5cm) was removed from its intestinal tract after anaesthesia. Secondly, the front of the pipette (200μl) was cut off by approx. 5 mm to reduce shear forces and then, after blowing out the intestinal tissue several times with the pipette (approx. 2min), the cell suspension was obtained by filtering the cell debris.

### Quality control of single cell suspension

**Figure 2:**
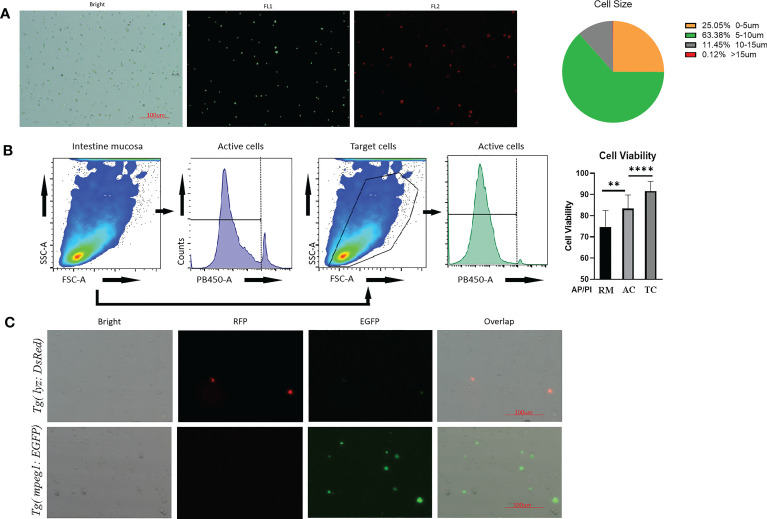
Quality control of prepared intestinal single cell suspension from *Tg(lyz:DsRED2)*;*Tg(mpeg1:EGFP)*. **(A)** Imaging by automated cell counter, cell under brightfield and fluorescence channels (FL1: 488nm; FL2:558nm). Scale bar: 100μm. The cellular diameter of the prepared intestinal mucosal cell suspension was calculated besides. **(B)** Cell viability reflected AO/PI staining during cytometric analysis. The stained cell number was counted by automatic cell counter. After AO/PI staining, 75% cell obtained from robbing method (RM) was alive, meanwhile for the blowing method, 80% of all cells (AC) and 90% of genetically fluorescence labeled immune cells, which were the target cells (TC), were alive for flow cytometric analysis. **(C)** Images of intestinal mucosal cells from *Tg(lyz:DsRED2)*;*Tg(mpeg1:EGFP)*. In single cell suspensions, DsRed labeled lyz^+^ cell and EGFP labeled mpeg1^+^ cells could be clearly observed, together with other unstained cells (shown in the brightfield). ** represented *p* < 0.01, **** represented *p* < 0.0001.

The round cells in the intestinal cell suspension were counted using an automated cell counter (**Figure 2A**). Most cells had diameters ranging from 5 to 15μm. There were around 25% of cells with diameters greater than 5μm, and only 0.12% of cells with diameters greater than 15μm (**Figure 2A**). As a result, the residual of cell debris and clumps was minimal. Notably, both flow cytometry and an automatic cell counter revealed that the vitality of intestinal cell suspension generated using the current blowing approach was 70%-85%. (**Figure 2B**). Using the population features of zebrafish immune cells (such as lymphocytes and neutrophils) (46, 47), we chose the gate of common target cells in the FSC-A and SSC-A channels for further investigation. Cell viability increased significantly, with cells in the selected gates having vitality more than 90%. In addition to physiological properties, immune cells from present genetically labelled zebrafish retained significant fluorescence (**Figure 2C**), which is essential for FACS.

### Enriching intestinal immune cells *via* stripping the muscular layer

**Figure 3:**
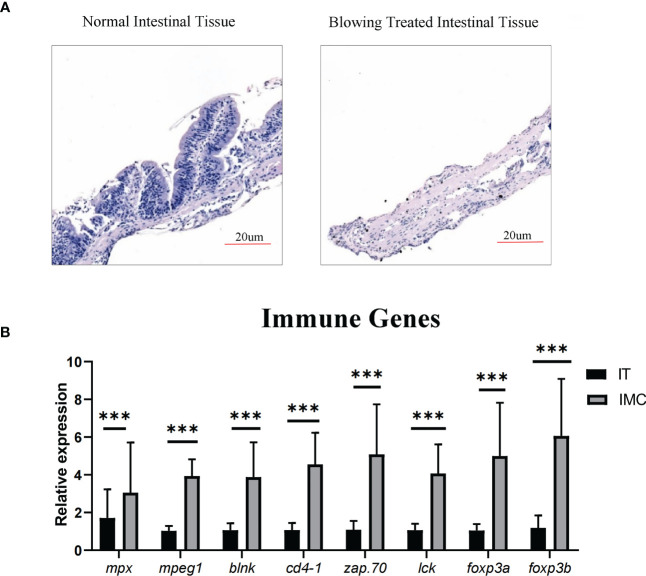
Enriching intestinal immune cells *via* stripping the muscular layer. **(A)** Morphological analysis of HE staining in comparison between intestinal tissue after blowing treatment and normal intestinal tissue. **(B)** qPCR analysis of genes involved in immune cell differentiation. All values are means ± SEM; statistical significance was determined by independent-samples T-test. For the comparison of IMC (intestinal mucosa cells) *vs* IT (intestinal tissue), *** represented p < 0.01.

According to HE stained zebrafish intestinal structure, the zebrafish gut consisted primarily of mucosa and muscularis, with the mucosa containing epithelium and lamina propria (**Figure 3A**). The current technique of blowing the intestinal epithelium has almost completely separated the mucosa from the muscle layer (**Figure 3A**). The cells in mucosal layers, including both intestinal epithelial layer and lamina propria, were then taken away by the PBS-FBS buffer (**Figure 3A**). Moreover, as shown in qPCR result (**Figure 3B**), the enrichment of immune cells in the cell suspension was also proved by the significantly increased (*P* < 0.05) expression of the immune genes, including markers of immune cells (neutrophil function related *mpx*, macrophages related *mpeg1*, B-cell receptor signaling related *blnk*, Th cell’s surface marker *cd4-1*, initiating T-cell responses related *zap70*, T cell activation related *lck*) and immune regulation related transcriptional factors (*foxp3a* and *foxp3b*).

### Enriched pathways, terms and cell makers for intestinal cell suspension

**Figure 4:**
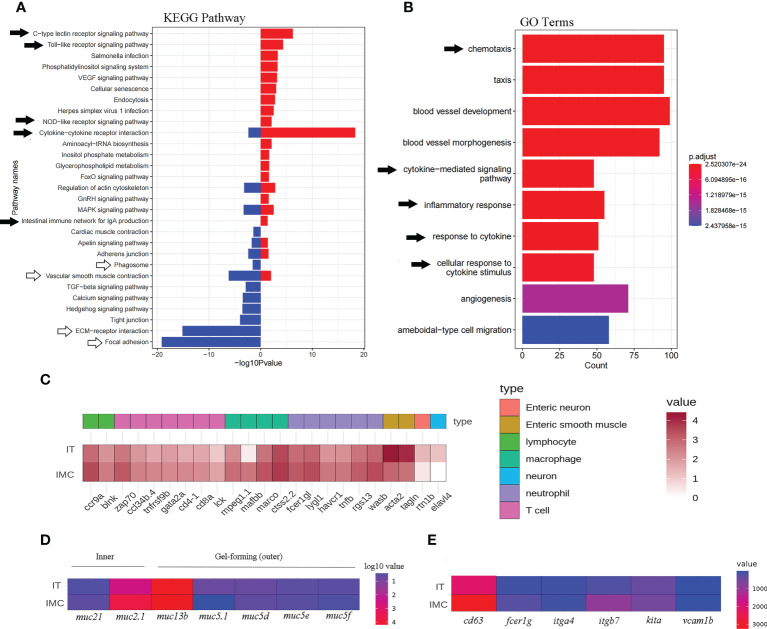
Enriched KEGG pathways and GO terms as well as heatmaps of mucosal immune related DEGs. Intestinal pathways **(A)** and terms **(B)** for the comparison between IMC and IT. **(C)** The heat map of immune cells maker genes. **(D)** The heat map of mucus related genes. **(E)** The heat map of mast cell related genes.

The pattern recognition-related “C-type lectin receptor signaling pathway,” “Toll-like receptor signaling pathway,” “NOD-like receptor signaling pathway,” as well as cytokine signaling-related “Cytokine-cytokine receptor interaction,” etc., were among the DEGs that showed an enrichment between the IMC and IT groups. Whereas barrier function-related KEGG pathways such as “vascular smooth muscle contraction,” “ECM-receptor interaction,” and “focal adhesion” were revealed for IT advantages (**Figure 4A**).

**Figure 5:**
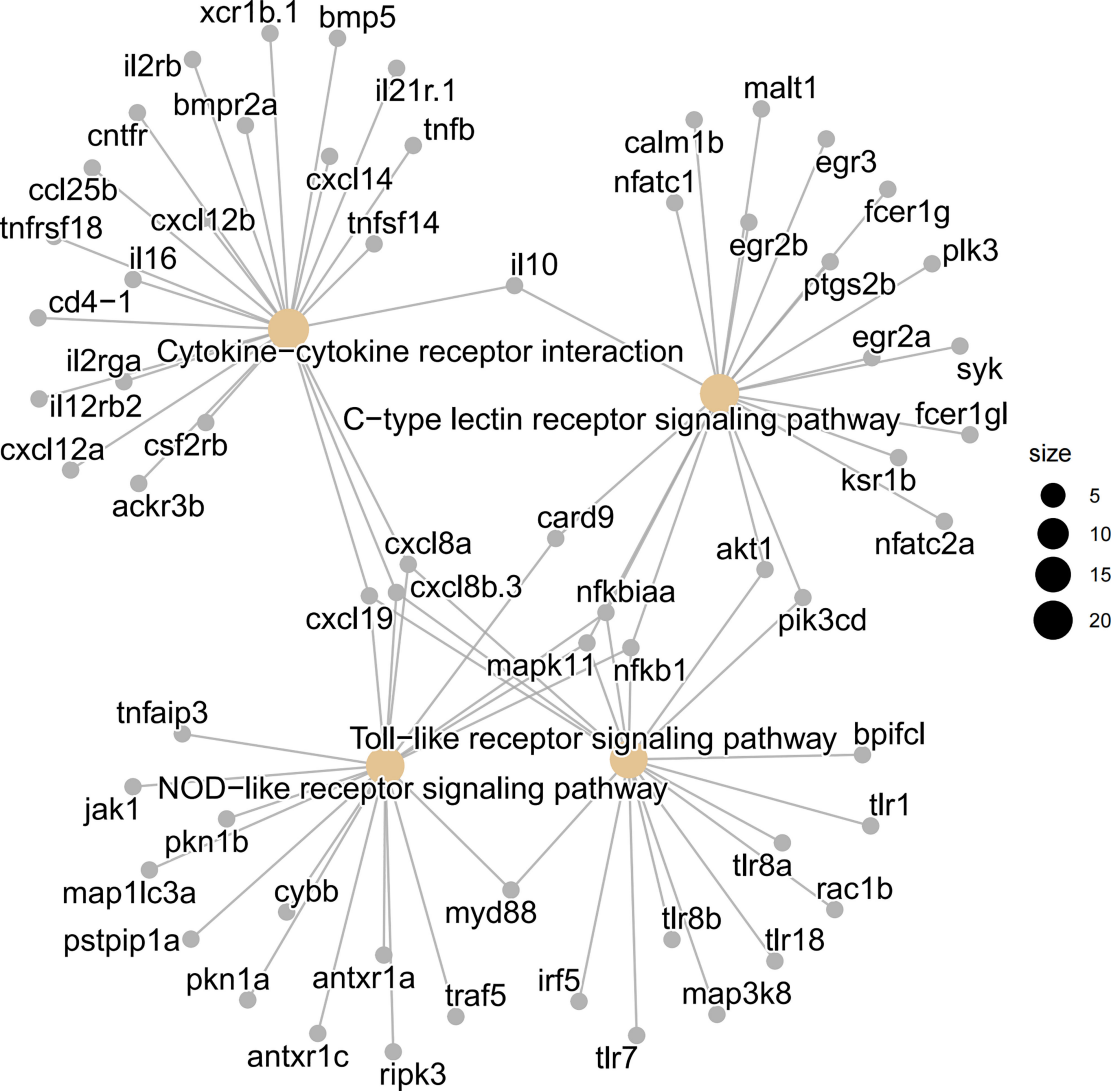
Visualization of involved genes in innate immune related KEGG pathways by cnetplot analysis. The pattern recognition related “C-type lectin receptor signaling pathway”, “NOD like receptor signaling pathway”, and “Toll like receptor signaling pathway”, as well as cytokine signaling related “cytokine-cytokine receptor interaction” were shown in details.

Regarding immune cell activity, a “phagosome” associated to macrophages was revealed for IT advantage while a B cell-related “intestinal immune network for IgA synthesis” was revealed solely for IMC benefit. Chemotaxis, cytokine-mediated signaling pathways, inflammatory responses, responses to cytokines, cellular responses to cytokine stimuli, etc. were among the immune-related enriched GO terms (**Figure 4B**). Regarding to the expression of cell type related genes, most immune genes were revealed for IMC advantage, while marker genes of neuron and muscle were IT advantage (**Figure 4C**). Meanwhile, the inner mucus related genes *muc2.1* and *muc21* were IMC advantage, yet the gel-forming (outer) mucus related genes *muc13b*, *muc5.1*, *muc5d*, et al. were IT advantage (**Figure 4D**). In addition, the mast cell’s marker genes *cd63*, *fcer1g1*, *itga7*, *kita*, et al. were IMC advantage (**Figure 4E**). Specifically, as to genes involved in innate immune related pathways, the pathogen sensing and chemotaxis related genes, such as *tlr*, *fcer*, *ccl*, and *cxcl* were IMC advantage (**Figure 5**).

### Gathered immune cell in intestinal cell suspension

**Figure 6:**
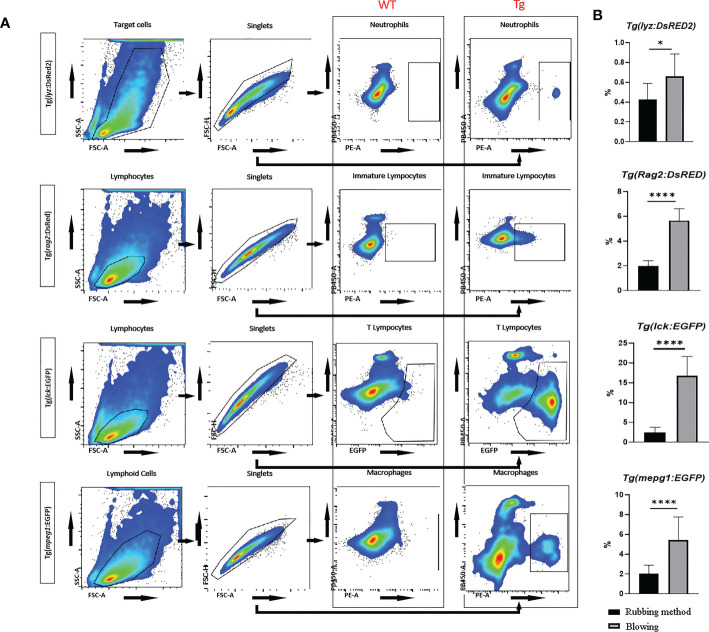
Flow cytometric analysis of transgenic zebrafish, including *Tg(lyz:DsRED2)*, *Tg(rag2:DsRed)*, *Tg(lck:EGFP)*, and *Tg(mpeg1:EGFP)*. **(A)** The gating strategies in cytometric analysis for intestinal neutrophils (with red fluorescence labeled Lyz), immature lymphocytes (with red fluorescence labeled Rag2), mature T lymphocytes (with green fluorescence labeled Lck), and macrophages (with green fluorescence labeled Mpeg1) in zebrafish. Compare with wild type samples, all types of fluorescence labeled immune cells could be accurately sorted. **(B)** Comparing samples made by mechanical dissociation of whole intestine and blowing off mucosal cells from muscularis, the fluorescence labeled cells were significantly enriched. * represented *p* < 0.05, **** represented *p* < 0.0001.

The aggregation of immune cells in the recently generated intestinal cell solution was examined using flow cytometry. Neutrophils, macrophages, immature lymphocytes, and T lymphocytes that were genetically fluorescently marked could be distinguished in significant numbers (**Figure 6A**). The cells from transgenic zebrafish have a completely distinct fluorescence expression profile from the wild type fish sample, and they can be specifically distinguished by flow cytometry in subgroups. Also, compared to the way of just rubbing tissue on the mesh, the proportion of immune cells from the present blowing method was significantly higher (**Figure 6B**).

### Checking tissue distribution of immune cells with current improved method

**Figure 7:**
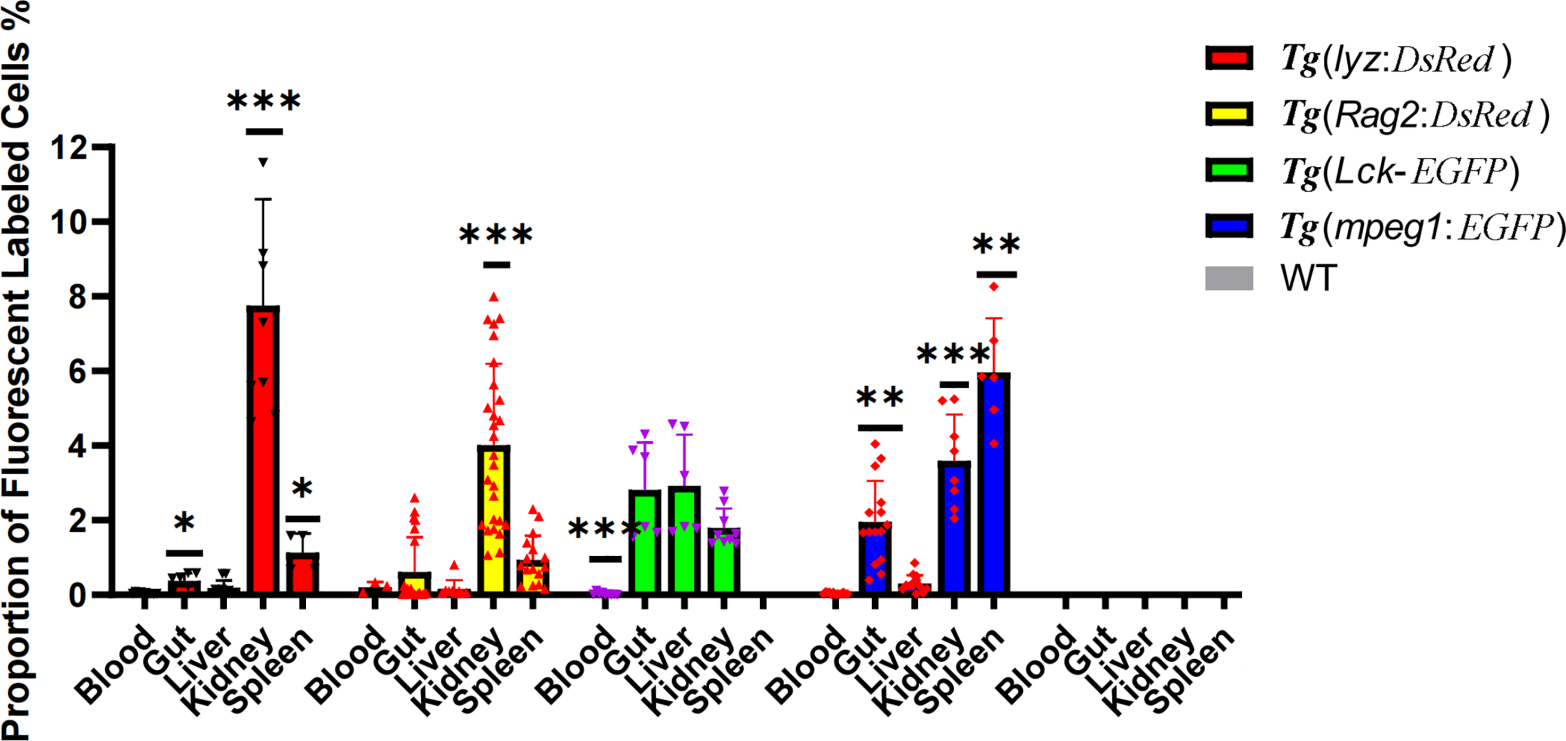
Proportion of fluorescent labeled immune cells, including neutrophils, macrophages, lymphocytes, and activated T cells, in immune organs (the periphery blood, intestine, liver, kidney and spleen). * represented *p* < 0.05, ** represented 0.01< p < 0.05. and *** represented p < 0.01.

As controls, systemic immune organs in zebrafish were evaluated in parallel with intestine samples obtained using the optimized approach. The proportion of lyz^+^ cells and Rag2^+^ cells in the kidney and spleen was found to be relatively high, but only detectable in the gut. Nevertheless, Lck^+^ T cells were detected in approximately equal numbers in the gut, liver, and kidney. Meanwhile, mpeg+ macrophages were found in large concentrations in the spleen, kidney, and gut. In particular, the intestinal cell suspension had a significant concentration of activated T cells (~3%), as well as macrophages (2%). Non-fluorescence labelled controls were also performed on wide type zebrafish cell suspension samples (**Figure 7**).

### The analysis of immune cells reflected inflammation in zebrafish gut

**Figure 8:**
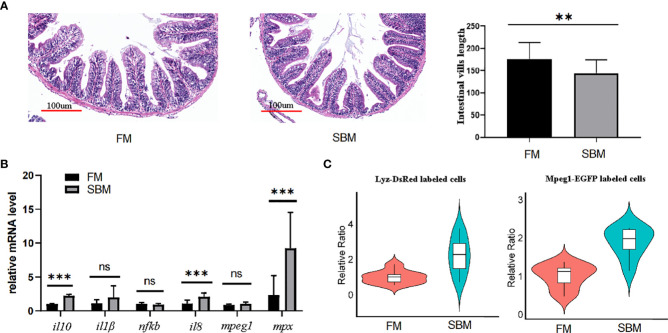
The SBMIE induced mucosa pathology was related to the altered composition of immune cells. The single cell suspension, obtained using current optimized method, accurately reflects altered composition of intestinal immune cells upon soybean induced inflammation. **(A)** Validation of inflammation by pathological analysis of intestinal mucosa. The length of intestinal villi was reduced at the condition of SBM (soybean meal) feeding. **(B)** qPCR analysis of genes involved in intestinal inflammation. **(C)** the increased proportion of intestinal mpeg^+^ and lyz^+^ cells upon SBMIE. *** represented p < 0.01. ns, no significance.

To test the pathological use of the current blowing approach for creating intestinal cell suspension, SBMIE modelling was used to induce aggregation of intestinal macrophages and neutrophils, which was then released by the blowing method. HE staining confirmed the pathophysiology of the middle intestine due to intestinal inflammation. Condensed blue spots in the LP layer revealed immune cell aggregation, and intestinal villi length was considerably (*p* = 0.0295) shorter in the FM group (**Figure 8A**). Further, the qPCR result of inflammation related genes showed that cytokine genes *il8* and *il10* was significantly upregulated, and proinflammatory factors *il1* and *nfkb* was also with the trend of upregulation in soybean (SBM) group against the fish meal (FM) group (**Figure 8B**). Meanwhile, the expression of cell maker genes *mpeg1* and *mpx* rose considerably in the SBM group (**Figure 8B**). At the cellular level, the proportions of macrophages and neutrophils in the intestinal cell suspension were considerably higher in the SBM group compared to the FM group (**Figure 8C**).

## Discussion

We have developed and made available a protocol for the preparation of intestinal cell suspensions for the analysis of immune cells in zebrafish of the strain AB. Flow cytometry was used to identify different types of immune cells, including neutrophils, macrophages and lymphocytes. The omics data also matched the histological and cellular results. Therefore, the current manipulation successfully isolated immune cells from the intestinal mucosa.

In teleost fish, the lamina propria is directly attached to the smooth muscle layers of the fish (48) due to the absence of the structural equivalents for submucosa and muscularis mucosa, which separates the lamina propria from the submucosa (35). As a model organism, the intestinal epithelium of the zebrafish was even thinner compared to other commercially available species (38), such as the common carp (49), grass carp (39) and tilapia (50). To avoid cell death, sample preparation must be completed and the cells diluted as quickly as possible. The fact that only 10 minutes are needed to prepare a single cell suspension for in-depth analysis (such as cytometric and omics studies) could guarantee the retention of physical and cellular properties. Among the intestinal cells currently isolated, the larger cells with a diameter of 10 to 15μm are likely to be the macrophages (51). Since the viability of the cells was over 90%, the high quality of the isolated cell suspension from the zebrafish intestinal mucosa may actually reflect physiology (50) and have greater functional capacity to support the study of the molecular mechanism by *in vitro* cell culture.

In suspension, the immune cells just released were mainly from the intestinal mucosa. The intestinal mucosa of the zebrafish consists of epithelium and lamina propria, which contain the majority of the immune cells in the intestine. As shown by both the HE staining and the DEGs, the main finding between the samples produced by the current blowing method and the whole intestine was the detachment of the muscle layer. Therefore, the cells in the suspension produced with the blowing method cannot be intestinal myocytes. At the same time, it was found that mucins, which are indicators of an acute gut stress response in fish (52), were also significantly altered after the cell suspension was prepared using the blowing method. Manipulation of the blowing increased the expression of membranous mucins associated with the inner mucus (53, 54), such as Muc2 and Muc21, but attenuated the abundance of mucins associated with the gel-forming mucus (55), such as Muc5.1. Since IL10 improves the characteristics of the mucus layer, SBMIE-induced upregulation of IL10 expression may contribute to more viscous intestinal samples (56). The removal of muscle and mucus from such connective tissue was critical for manipulating single cell suspensions. As a result, the present blowing procedure could efficiently isolate immune cells.

Regarding the immune aspect, immune cells were released sufficiently in both the IEL and LP layers. This was evidenced by the comparison of immune cells between different organs, including blood, kidney, spleen, liver and intestine. The ratio between immune cells from the intestine and other tissues was similar to previous reports (57, 58). In the enrichment analysis of DEG, the signaling pathways and terms related to the innate immune system were found with advantage for the intestinal mucosa cells (IMC) group. The pattern recognition, cytokine and chemokine signaling pathways and terms revealed indicate that IMC are more sensitive and immediate to stimulation, as the gastrointestinal tract is the interface between the host and the external environment (59). As indicated by the detailed network of KEGG pathways and GO terms, chemotaxis function was found to be advantageous in IMC. This is consistent with the fact that intestinal mast cells are a potent source of several chemokines (60) and zebrafish mast cells possess an FcεRI-like receptor and are involved in innate and adaptive immune responses (60). However, the IT advantage of the “phagosome” pathway may coincide with the lack of muscle macrophages in the IMC (61). Therefore, the currently prepared immune cell suspension is not suitable to study the local crosstalk between enteric neurons and macrophages (12).

Intestinal lymphocytes in particular play an important role in both tolerance and regulation. Since IgT (the fish equivalent of IgA) is a mucosal specialized Ig and could respond during inflammation, the enriched KEGG pathway “Intestinal immune network for IgA production” in the IMC group could reflect the response of mucosal B cells (62) and γδ-T cells (46). The fact that the amount of T lymphocytes isolated from the intestinal mucosa was comparable to the amount of T lymphocytes isolated from the kidney (the central immune organ of the fish) is consistent with the quantitative analysis of tissue-associated lymphocytes (63) performed with the currently developed method for isolating zebrafish mucosal immune cells.

Furthermore, the cytometric data showed that this blowing method can be used to analyze the intestinal response at the immune cell level. The increased ratio of mpeg^+^ and mpx^+^ cells in the gut of zebrafish after 3 months was consistent with previous results in the SBMIE model for zebrafish larvae (21–23). Cytometric analysis of the SBMIE of Atlantic salmon revealed that the immune cells in the whole blood were expended (64). Rapid and effective preparation of gut cell suspension containing almost all types of immune cells without consuming much reagents and time, such as mechanical dissociation and enzyme digestion, could be a viable protocol.

In summary, based on the characteristics of the zebrafish intestine, we have proposed an innovative method for the preparation of single-cell suspensions of zebrafish intestinal mucosa. The single-cell suspensions produced using the protocol we developed can promptly and accurately reflect the composition of the intestinal immune cells. In the future, more genetically labelled lines could be used to study the gut immune cell response with more detailed cell types. Considering that zebrafish enteritis models are also used to study human inflammatory bowel disease (19), the current blowing method and cytometric single mucosal cell suspension analysis protocol could facilitate both medical and aquaculture studies to understand the gastrointestinal immune mechanism.

## Data availability statement

The data presented in the study are deposited in the Genome Sequence Archive (GSA) database (http://gsa.big.ac.cn/index.jsp) with the BioProject identifier PRJCA015574.

## Ethics statement

The animal study was reviewed and approved by the Animal Research and Ethics Committees in the Institute of Hydrobiology, Chinese Academy of Sciences. Written informed consent (No. IHB2022-01) was obtained from the owners for the participation of their animals in this study.

## Author contributions

NW received the projects. JX, YL, XZ, LZ, LS, GW, and YC performed the experiments. XZ, YL, JX, QZ and NW wrote the manuscript. XZ, CG, HL, LZ and WZ did data analysis. X-QX revised the manuscript. All authors contributed to the article and approved the submitted version.
